# Body mass index and weight loss as risk factors for poor outcomes in patients with idiopathic pulmonary fibrosis: a systematic review and meta-analysis

**DOI:** 10.1080/07853890.2024.2311845

**Published:** 2024-02-01

**Authors:** Xing He, Jiaqi Ji, Chi Liu, Zeli Luo, Jialong Tang, Haiying Yan, Lu Guo

**Affiliations:** a Department of Pulmonary and Critical Care Medicine, Sichuan Provincial People’s Hospital, School of Medicine, University of Electronic Science and Technology of China, Chengdu, Sichuan Province, China; b Department of Pulmonary and Critical Care Medicine, Cheng Du Qing Cheng Mt. Hospital, Chongzhou City, Chengdu, Sichuan Province, China; c Department of Nephrology, Sichuan Academy of Medical Science and Sichuan Provincial People’s Hospital, Sichuan Renal Disease Clinical Research Center, University of Electronic Science and Technology of China, Chengdu, Sichuan Province, China; d Department of Critical Care Medicine, Wenjiang District People’s Hospital, Chengdu, Sichuan Province, China; e Department of Respiratory and Critical Care Medicine, Jiange County People’s Hospital, Guangyuan, Sichuan Province, China

**Keywords:** Idiopathic pulmonary fibrosis, body mass index, weight loss, mortality, acute exacerbation, hospitalization

## Abstract

**Objective:**

The association between nutritional status and prognosis of idiopathic pulmonary fibrosis (IPF) remains unclear. This systematic review and meta-analysis aimed to explore the effect of body mass index (BMI) and weight loss on the prognosis of IPF patients.

**Methods:**

We accumulated studies on IPF, BMI, and weight loss from databases including PubMed, Embase, Web of science, Scopus, Ovid and Cochrane Library up to 4 August 2023. Using Cox proportional hazard regression model for subgroup analysis, hazard ratio (HR) and 95% confidence intervals (CI) for BMI in relation to mortality, acute exacerbation (AE), and hospitalization in IPF patients were calculated, and HR, odds ratio (OR), and 95% CI for weight loss corresponding to IPF patient mortality were assessed. Sensitivity analysis was peformed by eliminating every study one by one, and publication bias was judged by Egger’s test and trim-and-fill method.

**Results:**

A total of 34 eligible studies involving 18,343 IPF patients were included in the meta-analysis. The pooled results by univariate Cox regression analysis showed that baseline BMI was a predictive factor for IPF mortality (HR = 0.93, 95%CI = [0.91, 0.94]). Furthermore, the results by the multivariable regression model indicated that baseline BMI was an independent risk factor for predicting IPF mortality (HR = 0.94, 95%CI = [0.91, 0.98]). Weight loss was identified as a risk factor for IPF mortality (HR = 2.74, 95% CI = [2.12, 3.54]; OR = 4.51, 95% CI = [1.72, 11.82]) and there was no predictive value of BMI for acute exacerbation (HR = 1.00, 95% CI= [0.93, 1.07]) or hospitalization (HR = 0.95, 95% CI = [0.89, 1.02]).

**Conclusion:**

Low baseline BMI and weight loss in the course of IPF may indicate a high risk of mortality in patients with IPF, so it is meaningful to monitor and manage the nutritional status of IPF patients, and early intervention should be conducted for low BMI and weight loss.

## Introduction

Idiopathic pulmonary fibrosis (IPF), as a chronic and progressive pulmonary disease, is characterized by irreversible deterioration of pulmonary function and has a short median survival time, accompanied by an age-standardized mortality rate ranging from 0.5 to 12 deaths per 100,000 individuals [1]. Factors such as lung function decline, acute exacerbation, and complications are related to IPF mortality [2, 3]. Recent studies have demonstrated that malnutrition and reduced food intake increase the risk of hospitalization and mortality in IPF patients, suggesting that nutritional status may have an adverse impact on the prognosis of IPF [4].

Body mass index (BMI) and weight change are important features of nutritional status, BMI has also been considered as a clinical indicator to assess malnutrition in IPF [5], while their significance in IPF patients has not been fully recognized, along with a lack of awareness in early intervention and management of nutritional status. Previous studies claimed that low BMI and weight loss predicted rapid lung-function decline [6], BMI may potentially serve as an indicator for predicting mortality, disease progression, and hospitalization risk in IPF patients, regardless of the use of antifibrotic drugs [7]. However no systematic quantitative analysis of the data was conducted [8], so the effect of nutritional status on the prognosis of IPF has not been fully elaborated.

Therefore, we performed this meta-analysis to reveal the clinical significance of BMI and weight loss in predicting mortality, acute exacerbation, and hospitalization in IPF patients through subgroup analysis by a qualitative approach.

## Methods

### Search strategy

The study was conducted in accordance with the Preferred Reporting Items for Systematic Reviews and Meta-Analyses (PRISMA) guidelines [9], and registered with INPLASY (http://INPLASY.com) under registration number INPLASY 2023110113. A comprehensive search for studies published leading up to 4 August 2023 was carried out from databases including PubMed, Embase, Web of science, Scopus, Ovid and Cochrane Library, with the following items: ‘idiopathic pulmonary fibrosis’, ‘body mass index’, ‘BMI’, ‘weight loss’, ‘body weight’, ‘mortality’, ‘exacerbation’ and ‘progression’ (Supplementary Table S1).

### Eligibility criteria

The inclusion criteria were as follows: (1) prospective or retrospective cohort studies; (2) IPF Diagnosis according to ATS/ERS/JRS/ALAT statement [10–12]: ①excluding other interstitial lung disease (ILD); ② chest high-resolution CT showing usual interstitial pneumonia changes, with assistance from multidisciplinary and lung biopsy if necessary; (3) hazard ratio (HR) and odds ratio (OR) analyzed by Cox proportional hazard model and logistic regression model respectively; (4) English studies.

The exclusion criteria were as follows: (1) Review/Meta-analysis, Case report/Letter, Conference Abstract; (2) other types of ILD; (3) inconsistent or unspecified statistical methods; (4) BMI or weight loss not studied as outcomes; (5) inability to extract data.

### Quality assessment (risk of bias) and data extraction

Two investigators (XH and JJ) critically screened studies independently, and if there was any disputation, a third investigator (LG) could be consulted. Newcastle-Ottawa Quality Assessment Scale (NOS) was used to evaluate the quality of studies [13], which evaluating the scores of the three dimensions of selection, comparability, and outcome of the included studies, the NOS total score was 9 ‘stars’: 0–3 ‘stars’ were low quality studies, 4–6 ‘stars’ were moderate quality, 7–9 ‘stars’ were high quality. Data was extracted, covering the listed items: the first author, year of publication, country, study type, study object, sample size, age, gender, antifibrotic agents, lung function, HR and 95% CI for baseline BMI as well as HR, OR and 95% CI for weight loss(weight loss was defined as annualized percent decline in body weight more than 0). Study Events covered mortality, acute exacerbation, and hospitalization.

### Data synthesis

Pooled HRs and ORs with 95% CIs were utilized to assess the predictive significance of BMI and weight loss for IPF mortality, acute exacerbation, and hospitalization. Heterogeneity was tested by Cochran’s Q statistic and inconsistency value (I^2^) [14]. If *p* < 0.05 or I^2^≥ 50%, it means remarkable heterogeneity, and the Dersimonian-Laird method should be applied to pool the data; otherwise, inverse-variance method would be chosen. Subgroup analysis was performed based on the type of Cox proportional hazard regression (univariate and multivariable). The sensitivity analysis [15] was calculated by eliminating every study one by one. The pooled result was considered stable and reliable if the exclusion of a category did not observably affect the result. Publication bias was judged by Egger’s test, and if *p* < 0.05, the impact of possible missing studies on the overall results was assessed using a nonparametric trim-and-fill method to determine the robustness of our results [16]. Statistical analysis was performed by Stata software (package meta, version 16.0) and *p* < 0.05 was considered statistically significant.

## Result

### Included studies

**Figure 1.**
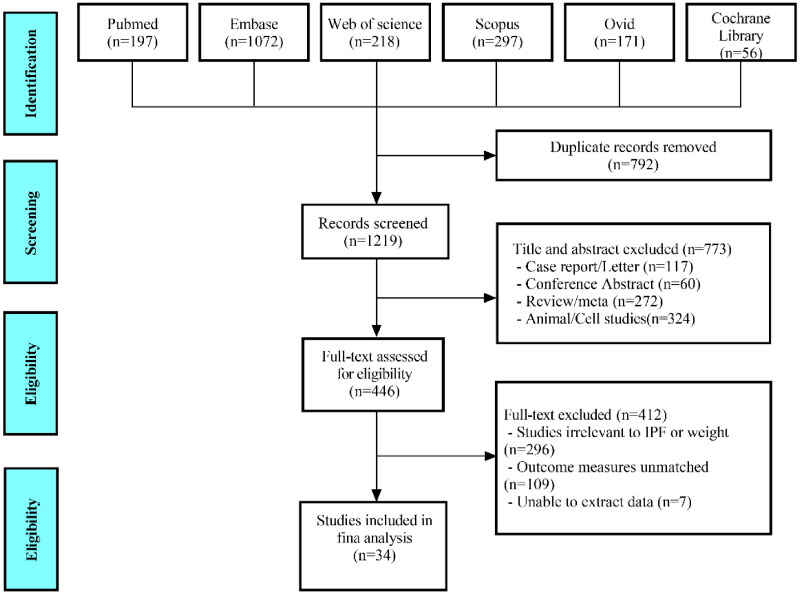
Diagram of the preferred reporting items for systematic review and meta-analysis (PRISMA).

Of 2011 studies identified after a comprehensive search, 34 eligible studies involving 18,343 IPF patients were enrolled in the meta-analysis based on careful examination of titles, abstracts, and full texts, which met the requirement of PRISMA statement (Figure 1). All studies were executed in different countries, covering Japan(*n* = 16), South Korea(*n* = 2), France(*n* = 4), Sweden(*n* = 1), Saudi Arabia(*n* = 2), Australia(*n* = 1), America(*n* = 4), China(*n* = 1), Canada(*n* = 1), Germany(*n* = 1) and Spain(*n* = 1). Among them, the predictive value of BMI in IPF patient was analyzed in 26 studies for mortality risk [4, 17–41](5 prospective studies, 21 retrospective studies), in 5 studies for acute exacerbation [26, 40–43](all retrospective studies) and in 3 studies for hospitalization [4, 44, 45](all retrospective studies); meanwhile, the predictive value of weight loss in IPF patient was investigated in 3 studies for mortality risk (HR) [18, 37, 46] (all retrospective studies), in 3 studies for mortality risk (OR) [7, 47, 48] (all retrospective studies). More details of each included study are displayed in Supplementary Tables S2–S6. With NOS score, 31 studies were considered to be of high quality and 3 were of moderate quality, indicating a potential risk of bias (Supplementary Table S7).

### Meta-analysis results

#### Hazard ratio of BMI predicting mortality

**Figure 2.**
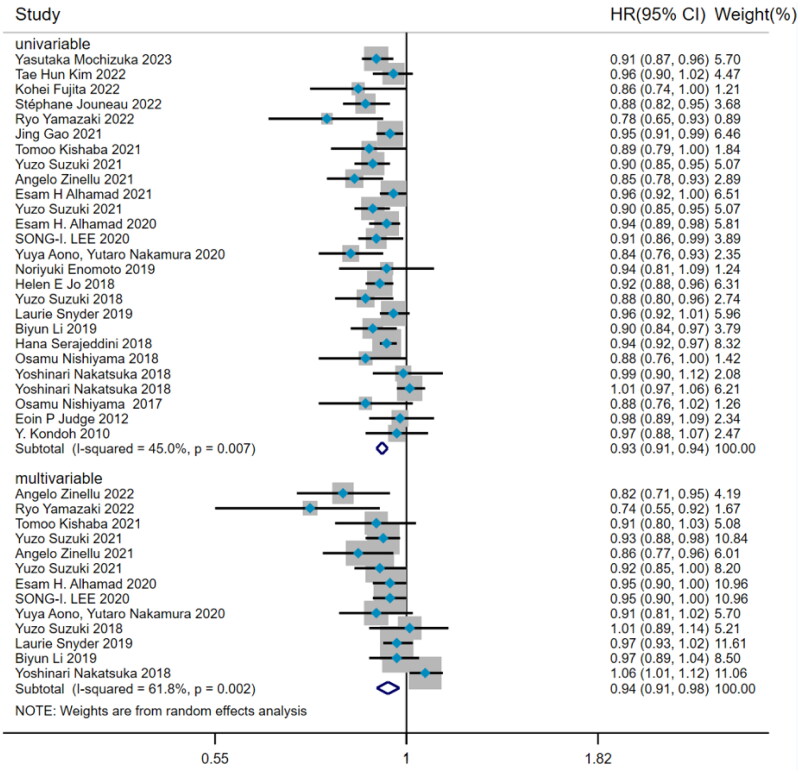
The forest plot pooled the hazard ratio of BMI predicting mortality in IPF.

There was noteworthy heterogeneity (I^2^=53.5%, *p* < 0.001) among the 26 studies about the predictive value of BMI for mortality risk in IPF patients. Subgroup analysis was performed by the type of Cox hazard regression. The results from 26 studies by univariate Cox regression presented that BMI was a risk factor for predicting mortality in IPF patients (HR = 0.93, 95%CI= [0.91, 0.94], *p* < 0.001) (Figure 2), with the method of DerSimonian–Laird. After adjusting confounding factors, the pooled analysis of 13 studies by multivariate Cox regression also proved that baseline BMI was an independent risk factor for predicting IPF mortality (HR = 0.94, 95%CI= [0.91,0.98], *p* = 0.001) (Figure 2).

#### Hazard ratio of BMI predicting acute exacerbation

**Figure 3.**
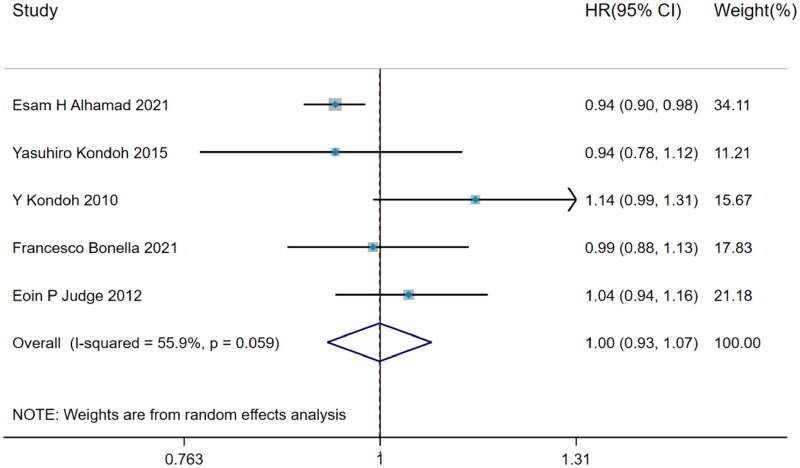
The forest plot pooled the hazard ratio of BMI predicting acute exacerbation in IPF.

A total of 5 studies with remarkable heterogeneity (I^2^=55.9%, *p* = 0.059) showed that BMI did not predict the acute exacerbation in IPF (HR = 1.00, 95%CI= [0.93,1.07], *p* > 0.05) (Figure 3), which was estimated by DerSimonian–Laird method.

#### Hazard ratio of BMI predicting hospitalization

**Figure 4.**
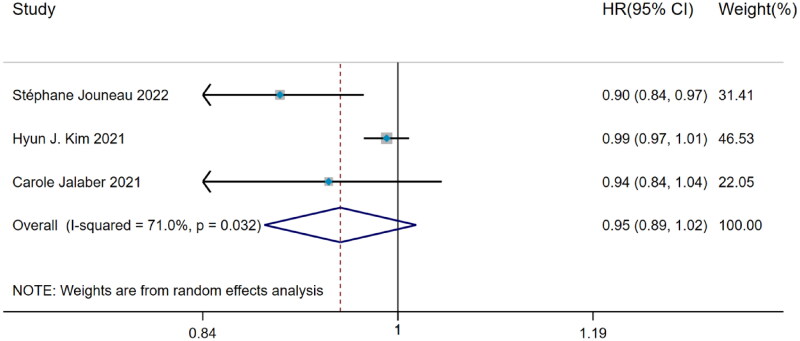
The forest plot pooled the hazard ratio of BMI predicting hospitalization in IPF.

Among 3 studies about the predictive value of BMI for hospitalization in IPF patients, significant heterogeneity was observed (I^2^=71.0%, *p* = 0.032). The DerSimonian–Laird method was used and pooled analysis came to the result that BMI had no predictive value for IPF hospitalization (HR = 0.95, 95%CI= [0.89, 1.02], *p* > 0.05) (Figure 4).

#### Odds ratio of weight loss predicting mortality

**Figure 5.**
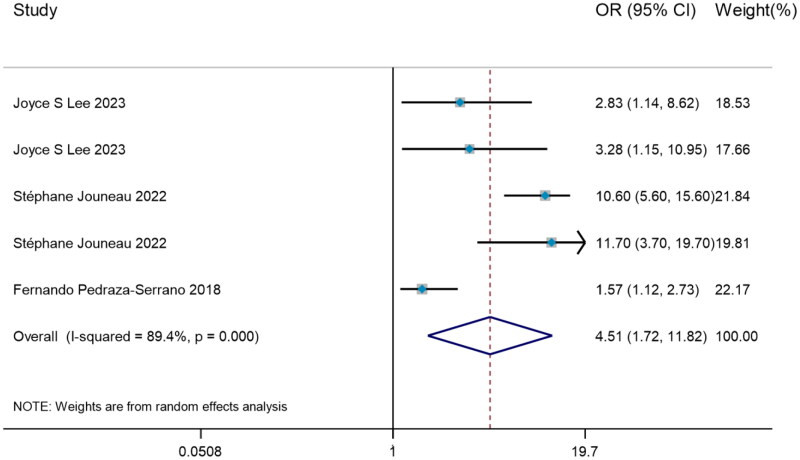
The forest plot pooled the odds ratio of weight loss predicting mortality in IPF.

To investigate how weight loss predicted the mortality risk in IPF, all 3 studies with significant heterogeneity (I^2^=89.4%, *p* < 0.001) were evaluated by DerSimonian–Laird method, with the result that weight loss may serve as a risk factor for predicting mortality in IPF (OR = 4.51, 95%CI= [1.72, 11.82]) (Figure 5).

#### Hazard ratio of weight loss predicting mortality

**Figure 6.**
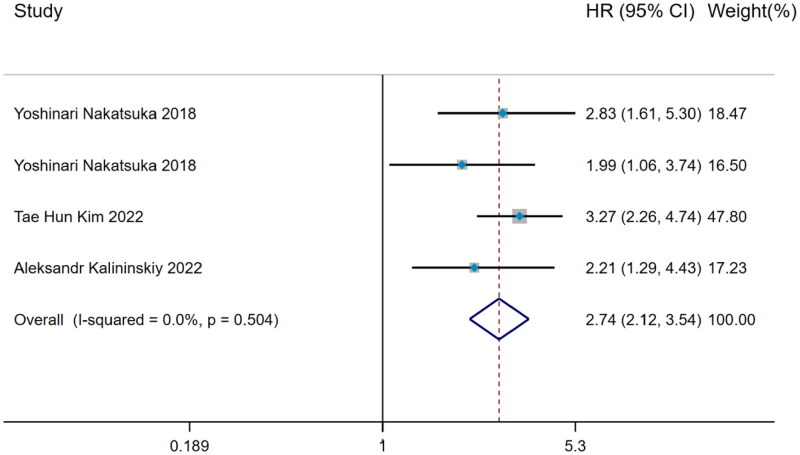
The forest plot pooled the hazard ratio of weight loss predicting mortality in IPF.

Including time variable to further explore the prediction of mortality risk in IPF patients with weight loss, the data was **calculated by** Inverse-Variance method on account of no significant heterogeneity in 3 relevant studies (I^2^ = 0%, *p* = 0.504) and the pooled analysis showed that weight loss was still a risk factor for predicting mortality risk in IPF (HR = 2.74, 95%CI= [2.12,3.54]) (Figure 6).

#### Sensitivity analysis and publication bias

**Figure 7.**
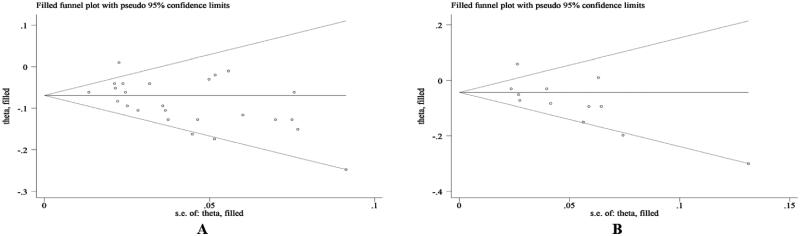
Funnel plots of trim-and-fill analysis (A: univariable; B: multivariable) for hazard ratio predicting mortality in IPF.

It pointed out that our results were stable by sensitivity analysis (Supplementary Figures S1–S5). The Egger’s test indicated potential publication bias in included studies referring to the predictive value of BMI for IPF mortality (both univariate and multivariable *p* < 0.05) (Table 1). Subsequently, trim-and-fill method was selected for adjustment, and the funnel plot and statistical results did not change, suggesting that our results was robust (Figure 7).

**Table 1.** Egger’s test for studies on BMI and weight loss predicting mortality, acute exacerbation and hospitalization in patients with IPF.

Group	nStudy	t	*P*
BMI and mortality(Univariate)	26	−2.54	0.018
BMI and mortality(Multivariable)	13	−2.37	0.037
BMI and acute exacerbation	5	1.66	0.195
BMI and hospitalization	3	−1.74	0.331
HR for weight loss and mortality	4	−2.69	0.115
OR for weight loss and mortality	5	0.39	0.721

## Discussion

Our results suggested that low BMI and weight loss were reliable markers for predicting the mortality risk in IPF, which means that improving BMI and weight loss can bring benefits to patients. To our knowledge, this is the first meta-analysis focusing on the effect of BMI and weight loss on the prognosis in IPF.

IPF is a disease with similar biological characteristics with cancer [49]. As the progression of pulmonary fibrosis, IPF patients gradually have a high risk of mortality. Lung transplantation is the only option to radically improve patients’ quality of life and prognosis, but only part of IPF patients can benefit from it due to various factors [50].

Anti-fibrotic medications can prolong median survival time in IPF to a certain extent and reduce the risk of mortality [51], but they cannot transform the ultimate outcome, so how to extend the survival of patients with IPF is a meaningful challenge. A latest study stated that nutrition, a topic without widespread concern, played a potential role in IPF mortality, and BMI and weight loss could increase the mortality rate of IPF [4]. However, this study lacked objectively pooled analysis and the impact of nutritional status on acute exacerbation and hospitalization of IPF is poorly understood [52]. Jouneau et al. described that reduced food intake and malnutrition at the time of IPF diagnosis were associated with hospitalization and mortality in IPF patients [4], indicating that low BMI and weight loss in patients may be important clinical signals for poor prognosis in IPF.

In our study, the results by univariate Cox regression firstly demonstrated the ability of BMI to predict the mortality risk in IPF. Considering confounding factors such as age, gender, smoking history, pulmonary function, and anti-fibrosis drugs, we further analyzed data by multivariate Cox regression, which ultimately confirmed the consistency with the results of the univariate analysis. It is believed that low BMI is more likely to induce lung infections and acute exacerbations [53, 54], leading to high mortality risk. On the other hand, weight loss not only reflects the deterioration of nutritional status and disease progression in IPF patients, but also acts as a risk factor for predicting IPF mortality [37]. It is necessary to pay attention to factors related to BMI and weight loss in IPF patients, such as anti-fibrosis drugs with adverse reactions including decreased appetite and diarrhea [55]. In addition, patients with IPF suffer from varying degrees of chronic hypoxia, which further suppresses appetite by regulating certain hormones, resulting in reduced food intake [56]. Meanwhile, hypoxia also contributes to intestinal damage and inflammation [57], hindering the digestion and absorption of nutrients. Although theoretically improving the digestive function and nutritional status for patients may be benefit to IPF survival, its impact on IPF prognosis is not clear.

Our study found that BMI was not a risk factor for acute exacerbation of IPF (AE-IPF), which is in accord with the conclusion by Zinellu et al. [58]. AE-IPF may be triggered by various factors, including infection, gastroesophageal reflux, surgical trauma and so on [59]. The influence of BMI on AE-IPF is controversial. Low BMI level may reflect poor nutritional status and raise the risk of infection, as previously mentioned, while high BMI means obesity and increases the risk of gastroesophageal reflux [60]. Considering that low BMI enhances the mortality risk from AE-IPF and high BMI is associated with low mortality in AE-IPF patients [53], improving BMI may serve as a potential therapeutic strategy to reduce AE-IPF and lower the mortality risk. Additionally, BMI cannot be used as a marker for predicting hospitalization in IPF patients. Although low BMI is correlated with an increased risk of infection, acute exacerbations, and mortality, there are hospitalization indicators for these patients, and investigators could not confirm if there are other hospital admissions. Besides, patients with low BMI may be stable after outpatient management without the need for hospitalization. In conclusion, more large-scale prospective data is needed for predictive value of BMI for acute exacerbation and hospitalization risk in IPF patients.

Nutritional status is a prominent prognostic factor for patients with chronic pulmonary diseases and has been paid more and more attention in recent years. In patients with chronic obstructive pulmonary disease (COPD), low BMI means poor pulmonary function and high symptom scores, signifying a high risk of mortality and hospitalization [61]. However, the mortality rate of asthma patients with high BMI increases year by year [62], and the cumulative incidence of acute exacerbations boosts [63]. Therefore, it is necessary to enhance the monitoring of BMI and body weight of IPF patients, eliminate adverse reactions related to anti-fibrosis drugs, and provide individualized nutrition management strategies.

There are some limitations in our study. Firstly, we could not conduct a quantitative meta-analysis of BMI considering the lack of data, nor did we stratify BMI for subgroup analysis. Secondly, the effect of fat-free mass index and nutrition score on the IPF prognosis also needs to be considered. In view of a great many factors that may influence the prognosis of IPF, including the degree of pulmonary function impairment, comorbidities, and response to therapeutic drugs, our study could not eliminate the effect of these factors. Finally, most of the studies were univariate analyses, varied follow-up times, and small numbers of outcomes, which may reduce the precision of the findings, more studies are expected to clarify the relationship between weight loss and acute exacerbation as well as disease progression of IPF.

## Conclusion

Low BMI and weight loss play a predictive role in the mortality of IPF patients, indicating that nutritional status can affect patient prognosis. Monitoring changes in BMI and weight, and provide early and effective interventions, which may be vital strategies for improving IPF prognosis.

## Supplementary Material

Supplemental Material

## Data Availability

The original data presented in the study are included in the article/Supplementary Material, further inquiries can be directed to the corresponding author.
