# Supplementary material for: Body mass index and weight loss as risk factors for poor outcomes in patients with idiopathic pulmonary fibrosis: a systematic review and meta-analysis
Source: Ann Med. 2024 Feb 1;56(1):2311845. doi: 10.1080/07853890.2024.2311845 (PMC10836485; doi:10.1080/07853890.2024.2311845)

Supplementary Table 1. Database and Search strategy

| **Database** | **Search strategy** |
| --- | --- |
| **Pubmed** | #1: ((IPF[title/abstract] OR idiopathic pulmonary fibrosis[title/abstract]) ) OR (Idiopathic Pulmonary Fibrosis[MeSH Terms]) |
|  | #2: (((((exacerbation[Title/Abstract]) OR (progression[Title/Abstract])) OR (progressive[Title/Abstract])) OR (death[Title/Abstract])) OR (deceased[Title/Abstract])) OR (mortality[Title/Abstract]) |
|  | #3: ((((body weight[Text]) OR (weight loss[Text])) OR (BMI[Text])) OR (body mass index[Text])) OR (body weight[MeSH Terms]) |
|  | #4:#1 AND #2 AND #3 = **197** items |
| **Embase** | #1: 'fibrosing alveolitis'/exp OR 'IPF':ab,ti OR 'idiopathic pulmonary fibrosis':ab,ti |
|  | #2: 'death':ab,ti OR 'mortality':ab,ti OR 'die':ab,ti OR 'progressive':ab,ti OR 'progression':ab,ti OR 'exacerbation':ab,ti |
|  | #3: 'body weight'/exp OR 'BMI':ab,ti OR 'Body Mass Index':ab,ti OR 'body weight':ab,ti OR 'weight loss':ab,ti OR |
|  | #4:#1 AND #2 AND #3 = **1072** items |
| **Cochrane library** | #1: (idiopathic pulmonary fibrosis):MeSH OR (IPF):ab,ti,kw OR (idiopathic pulmonary fibrosis):ab,ti,kw |
|  | #2: (body weight):MeSH OR (BMI):ab,ti,kw OR (Body Mass Index):ab,ti,kw OR (body weight):ab,ti,kw OR (weight loss):ab,ti,kw |
|  | #3: (death):ab,ti,kw OR (mortality):ab,ti,kw OR (progressive):ab,ti,kwOR(progression):ab,ti,kwOR(exacerbation):ab,ti,kw |
|  | #4:#1 AND #2 AND #3 = **56** items |
| **Web of science** | #1: TS=(IPF OR idiopathic pulmonary fibrosis) |
|  | #2: TS=(BMI OR Body Mass Index OR body weight OR weight loss) |
|  | #3: TS=(death OR mortality OR progressive OR progression OR exacerbation) |
|  | #4:#1 AND #2 AND #3 =**218** items |
| **Scopus** | #1: TITLE-ABS-KEY ("IPF" OR "idiopathic pulmonary fibrosis") |
|  | #2: TITLE-ABS-KEY ( "BMI" OR "Body Mass Index" OR "body weight" OR "weight loss") |
|  | #3: TITLE-ABS-KEY ("death" OR "mortality" OR "progressive" OR "progression"OR "exacerbation") |
|  | #4:#1 AND #2 AND #3 =**297** items |
| **Ovid** | #1: (IPF OR idiopathic pulmonary fibrosis).ti,ab,kw |
|  | #2: (BMI OR Body Mass Index OR body weight OR weight loss).ti,ab,kw |
|  | #3: (death OR mortality OR progressive OR progression OR exacerbation).ti,ab,kw |
|  | #4:#1 AND #2 AND #3 =**171** items |

Supplementary Table 2 Characteristics of each study about hazard ratio of BMI predicting mortality of IPF

| **No.** | **Author** | **Time** | **Country** | **Study type** | **nTotal/**  **nDeath** | **Age**  **(year)** | **nMale** | **nPFD/**  **NTD** | **FVC%** | **DLCO%** | **BMI type** | **HR** | **Lower limit** | **Higher limit** | **Follow-up period** | Cox proportional models | Variables in model |
| --- | --- | --- | --- | --- | --- | --- | --- | --- | --- | --- | --- | --- | --- | --- | --- | --- | --- |
|  |  |  |  |  |  |  |  |  |  |  |  |  |  |  | (minimum/month) |  |  |
| 1 | Mochizuka Y[17] | 2023 | Japan | retrospective cohort | 301/300 | 72  [68-77]^*^ | 247 | 155/146 | 69.9  [59.1-81.8] | 61.0  [45.5-74.2] | continuous | 0.91 | 0.87 | 0.96 | 12m | univariable | BMI |
| 2 | Kim TH[18] | 2022 | South Korea | retrospective cohort | 215/213 | 71.8±7.4^#^ | 175 | 215/NA | NA | NA | continuous | 0.96 | 0.9 | 1.02 | NA | univariable | BMI |
| 3 | Fujita K[19] | 2022 | Japan | retrospective cohort | 61/21 | 70.1±7.9 | 54 | NA | 83.7  [69.4-92.5] | 73.0  [54.8-85.8] | continuous | 0.86 | 0.74 | 1 | 12m | univariable | BMI |
| 4 | Jouneau S[4] | 2022 | France | retrospective cohort | 153/52 | 72.4±8.1 | 119 | 58/38 | 81.7±17.5 | 45.5±16.8 | continuous | 0.88 | 0.82 | 0.95 | 12m | univariable | BMI |
| 5 | Zinellu A[20] | 2021 | France/ Italy | retrospective  cohort | 90/62 | 70.1±6.3 | 79 | 50/6 | 74.7  [61.6-89.3] | 42.3  [31.0-54.4] | continuous | 0.82 | 0.71 | 0.95 | 48m | multivariable | BMI、age、gender、smoking status、therapy |
| 6 | Yamazaki R[21] | 2022 | Japan | retrospective cohort | 107/NA | NA | 87 | NA | NA | NA | continuous | 0.78 | 0.65 | 0.93 | 33m | univariable | BMI |
|  |  |  |  |  |  |  |  |  |  |  |  | 0.74 | 0.55 | 0.92 | 33m | multivariable | BMI、age、gender、FVC%、DLCO%、therapy |
| 7 | Gao J[22] | 2021 | Sweden | prospective  cohort | 662/195 | 72.7  [68.0-78.0] | 490 | NA | 71.0  [61.0-85.0] | 47.0  [37.0-56.0] | continuous | 0.95 | 0.91 | 0.99 | 6m | univariable | BMI |
| 8 | Kishaba T[23] | 2021 | Japan | retrospective cohort | 39/24 | 72.9±7.0 | 27 | NA | 66.8±14.9 | 64.6±27.9 | continuous | 0.89 | 0.79 | 1 | 7m | univariable | BMI |
|  |  |  |  |  |  |  |  |  |  |  |  | 0.91 | 0.8 | 1.03 | 7m | multivariable | BMI、age |
| 9 | Suzuki Y[24] | 2021 | Japan | retrospective cohort | 229/122 | 72.0  [67.5-72.0] | 186 | NA | 68.3  [57.0-80.7] | 59.0  [44.4-71.3] | continuous | 0.9 | 0.85 | 0.95 | NA | univariable | BMI |
|  |  |  |  |  |  |  |  |  |  |  |  | 0.93 | 0.88 | 0.98 | NA | multivariable | BMI、history of AE、LTOT、GAP index |
| 10 | Zinellu A[25] | 2021 | France | retrospective cohort | 82/45 | 72±7 | 73 | 46/6 | 77.0±19.2 | 45.7±18.3 | continuous | 0.85 | 0.78 | 0.93 | 48m | univariable | BMI |
|  |  |  |  |  |  |  |  |  |  |  |  | 0.86 | 0.77 | 0.96 | 48m | multivariable | BMI、age、gender、smoking status、disease stage、AISI |
| 11 | Alhamad EH[26] | 2021 | Saudi Arabia | retrospective cohort | 204/NA | NA | NA | NA | NA | NA | continuous | 0.96 | 0.92 | 1 | 6m | univariable | BMI |
| 12 | Suzuki Y[27] | 2021 | Japan | retrospective cohort | 208/123 | NA | 176 | NA | NA | NA | continuous | 0.9 | 0.85 | 0.95 | NA | univariable | BMI |
|  |  |  |  |  |  |  |  |  |  |  |  | 0.92 | 0.85 | 1 | NA | multivariable | BMI、age、gender、ESM_CSA_、FVC%、DLCO% |
| 13 | Alhamad EH[28] | 2020 | Saudi Arabia | retrospective cohort | 212/68 | 66.4±11.7 | 150 | 40/20 | 53.7±20.0 | 43.0±20.5 | continuous | 0.94 | 0.89 | 0.98 | NA | univariable | BMI |
|  |  |  |  |  |  |  |  |  |  |  |  | 0.95 | 0.9 | 1 | NA | multivariable | BMI、AE、final saturation、therapy、6MWD、TLC%、FVC% |
| 14 | LEE SI[29] | 2020 | South Korea | retrospective cohort | 445/NA | 66.4±7.8 | 340 | NA | 68.7±15.7 | 54.9±16.2 | continuous | 0.91 | 0.86 | 0.99 | NA | univariable | BMI |
|  |  |  |  |  |  |  |  |  |  |  |  | 0.95 | 0.9 | 1 | NA | multivariable | BMI、age、FVC%、DLCO%、6MWD、SpO_2_、BALneu、BALlym、PPFE |
| 15 | Yutaro Nakamura YA[30] | 2020 | Japan | retrospective cohort | 105/NA | 70  [39-83] | 100 | 59/46 | 68.0  [33.6-132.6] | 52.6  [21.7-89.9] | continuous | 0.84 | 0.76 | 0.93 | 24m | univariable | BMI |
|  |  |  |  |  |  |  |  |  |  |  |  | 0.91 | 0.81 | 1.02 | 24m | multivariable | BMI、FVC、ΔFVC、therapy |
| 16 | Enomoto N[31] | 2019 | Japan | retrospective cohort | 39/23 | 69  [50-84] | 37 | NA | 57.6  [37.5-89.3] | 57.1  [33.5-85.9] | continuous | 0.94 | 0.81 | 1.09 | NA | univariable | BMI |
| 17 | Jo HE[39] | 2017 | Australia | prospective cohort | 416/22 | NA | NA | NA | NA | NA | continuous | 0.92 | 0.88 | 0.96 | 6m | univariable | BMI |
| 18 | Suzuki Y[34] | 2018 | Japan | retrospective cohort | 131/76 | 69.0  [64.0-75.0] | 117 | NA | 80.5  [66.4-92.9] | 68.6  [55.4-97.1] | continuous | 0.88 | 0.8 | 0.96 | NA | univariable | BMI |
|  |  |  |  |  |  |  |  |  |  |  |  | 1.01 | 0.89 | 1.14 | NA | multivariable | BMI、age、gender、ESM_CSA_、ESM_MA_、FVC% |
| 19 | Snyder L[32] | 2019 | America | prospective cohort | 662/92 | 70  [65-75] | 496 | NA | 69.6  [60.1-79.9] | 41.7  [32.2-50.1] | continuous | 0.96 | 0.92 | 1.01 | 30m | univariable | BMI |
|  |  |  |  |  |  |  |  |  |  |  |  | 0.97 | 0.93 | 1.02 | 30m | multivariable | BMI、age、gender、insurance、smoking status、oxygen use、comorbidity、prior hospitalisation、distance to enrolling centre、symptom onset |
| 20 | Li BY[33] | 2019 | China | retrospective cohort | 148/70 | NA | 133 | NA | NA | NA | continuous | 0.9 | 0.84 | 0.97 | NA | univariable | BMI |
|  |  |  |  |  |  |  |  |  |  |  |  | 0.9 | 0.84 | 0.97 | NA | multivariable | BMI、FVC%、serum albumin、serum globumin、serum PA |
| 21 | Serajeddini H[35] | 2018 | Canada | retrospective cohort | 437/105 | 64±9 | 314 | NA | 60±21 | 42±17 | continuous | 0.94 | 0.92 | 0.97 | 18m | univariable | BMI |
| 22 | Nishiyama O[36] | 2017 | Japan | prospective  cohort | 31/23 | 72.3±5.3 | 23 | NA | 73.8±18.3 | 61.0±16.7 | continuous | 0.88 | 0.76 | 1 | 45m | univariable | BMI |
| 23 | Nakatsuka Y[37] | 2018 | Japan/ Britain | retrospective cohort | 210/125 |  |  |  |  |  |  | 0.99 | 0.9 | 1.12 | 12m | univariable |  |
|  |  |  |  |  |  | NA | 174 | 55/NA | NA | NA | continuous | 1.01 | 0.97 | 1.06 | 12m | univariable | BMI |
|  |  |  |  |  |  |  |  |  |  |  |  | 1.06 | 1.01 | 1.12 | 12m | multivariable | BMI、weight loss、DLCO%、FVC decline>10% |
| 24 | Nishiyama O[38] | 2017 | Japan | prospective  cohort | 44/21 | 72.3±7.2 | 35 | NA | 82.6±20.8 | 69.3±19.3 | continuous | 0.88 | 0.76 | 1.02 | 20m | univariable | BMI |
| 25 | Judge EP[40] | 2012 | Japan | retrospective cohor | 55/22 | NA | 41 | NA | NA | NA | continuous | 0.98 | 0.89 | 1.09 | NA | univariable | BMI |
| 26 | Kondoh Y[41] | 2010 | Japan | retrospective cohor | 74/57 | 64.1±7.4 | 61 | NA | 77.0±19.2 | 59.37±18.7 | continuous | 0.97 | 0.88 | 1.07 | 55m | univariable | BMI |

*:Mean±standard deviation; ^#^:Median[interquartile range]; %: percentage of predicted value; NA: not applicable; PFD: pirfenidone; NTD: nintedanib; FVC: forced vital capacity; DLCO: diffusing lung capacity for carbon monoxide; GAP: GAP Gender-Age-Physiology; LTOT: long-term oxygen therapy; AISI: Aggregate Index of Systemic Inflammation; ESM_CSA:_ cross-sectional area of erector spinae muscles; ESM_MA_: muscle attenuation of elector spine muscles; AE: acute exacerbation; 6MWD: six minute walk distance; TLC: total lung capacity, SpO_2_: peripheral blood oxygen saturation; BAL: bronchoalveolar lavage; lym: lymphocyte; neu: neutrophil; PPFE: pleuroparenchymal fibroelastosis; ΔFVC: 6-month period of FVC change; PA: prealbumin

Supplementary Table 3 Characteristics of each study about hazard ratio of BMI predicting acute exacerbation of IPF

| **No.** | **Author** | **Time** | **Country** | **Study type** | **ntotal/nAE** | **Age**  **(year)** | **nMale** | **nPFD/**  **NTD** | **FVC%** | **DLCO%** | **BMI type** | **HR** | **Lower limit** | **Higher limit** | **Follow-up period**  **(minimum/month)** | **Cox proportional models** |
| --- | --- | --- | --- | --- | --- | --- | --- | --- | --- | --- | --- | --- | --- | --- | --- | --- |
| 1 | Alhamad EH[26] | 2021 | Saudi Arabia | retrospective cohort | 147/83 | NA | NA | NA | NA | NA | continuous | 0.94 | 0.9 | 0.98 | 6m | univariable |
| 2 | Kondoh Y[43] | 2015 | Japan | retrospective cohort | 267/14 | 64.8±6.9 | 213 | NA | 77.8±17.4 | 53.6±17.8 | continuous | 0.94 | 0.78 | 1.12 | 12m | univariable |
| 3 | Kondoh Y[41] | 2010 | Japan | retrospective cohort | 74/23 | 64.1±7.4 | 61 | NA | 77.0±19.2 | 59.3±18.7 | continuous | 1.14 | 0.99 | 1.31 | 55m | univariable |
| 4 | Bonella F[42] | 2021 | Germany | retrospective cohort | 62/19 | 63.5±11 | 43 | NA | 69±21 | 43±14 | continuous | 0.99 | 0.88 | 1.13 | NA | univariable |
| 5 | Judge EP[40] | 2012 | Japan | retrospective cohort | 55/27 | 60.04±7.51 | 41 | NA | 59.3±14.0 | 30.7±10.2 | continuous | 1.04 | 0.94 | 1.16 | NA | univariable |

PFD: pirfenidone; NTD: nintedanib; %: percentage of predicted value; FVC: forced vital capacity; DLCO: diffusing lung capacity for carbon monoxide; NA: not applicable

Supplementary Table 4 Characteristics of each study about hazard ratio of BMI predicting hospitalization of IPF

| **No.** | **Author** | **Time** | **Country** | **Study type** | **ntotal/**  **nhospitalization** | **Age**  **(year)** | **nMale** | **nPFD/**  **NTD** | **FVC%** | **DLCO%** | **BMI type** | **HR** | **Lower limit** | **Higher limit** | **Follow-up period**  **(minimum/month)** | **Cox proportional models** |
| --- | --- | --- | --- | --- | --- | --- | --- | --- | --- | --- | --- | --- | --- | --- | --- | --- |
| 1 | Jouneau S[4] | 2022 | France | retrospective cohort | 153/55 | 72.4±8.1 | 119 | 58/38 | 81.7%±17.5 | 45.4±16.8 | continuous | 0.9 | 0.84 | 0.97 | 12m | univariable |
| 2 | Kim HJ[45] | 2021 | America | retrospective cohort | 1002/568 | 71  [66-75] | 419 | NA | 69.2  [58.5-79.3] | 40.8  [30.9-49.8] | continuous | 0.99 | 0.97 | 1.01 | 67m | univariable |
| 3 | Jalaber C[44] | 2021 | Japan | retrospective cohort | 71/25 | 74.09±7.52 | 54 | NA | 81.1±17.5 | 46.0±13.7 | continuous | 0.94 | 0.84 | 1.04 | 14m | univariable |

PFD: pirfenidone; NTD: nintedanib; %: percentage of predicted value; FVC: forced vital capacity; DLCO: diffusing lung capacity for carbon monoxide; NA: not applicable

Supplementary Table 5 Characteristics of each study about odds ratio of weight loss predicting mortality of IPF

| **No.** | **Author** | **Time** | **Country** | **Study type** | **ntotal/ndeath** | **Age**  **(year)** | **nMale** | **nPFD/**  **NTD** | **FVC%** | **DLCO%** | **Weight loss****^&^**  **type** | **OR** | **Lower limit** | **Higher limit** | **Follow-up period**  **(minimum/month)** | **Cox proportional models** |
| --- | --- | --- | --- | --- | --- | --- | --- | --- | --- | --- | --- | --- | --- | --- | --- | --- |
| 1 | Lee JS[47] | 2023 | America | retrospective cohort | 365/65 | 71.3±7.8 | 324 | 163/131 | 70.5±16.8 | NA | categorical  >0 to <5% | 2.83 | 1.14 | 8.62 | 24m | univariable |
| 2 | Lee JS[47] | 2023 | America | retrospective cohort | 71/17 | 71.3±6.9 | 58 | 27/37 | 64.4±14.6 | NA | categorical  ≥5% | 3.28 | 1.15 | 10.95 | 24m | univariable |
| 3 | Jouneau S[7] | 2022 | France | retrospective cohort | 600/  118 | NA | NA | NA | NA | NA | categorical  >0 to <5% | 10.6 | 5.6 | 15.6 | 12m | univariable |
| 4 | Jouneau S[7] | 2022 | France | retrospective cohort | 99/45 | NA | NA | NA | NA | NA | categorical  ≥5% | 11.7 | 3.7 | 19.7 | 12m | univariable |
| 5 | Pedraza-Serrano F[48] | 2018 | Spain | retrospective cohort | 10285/NA | 73.7±11.0 | 5886 | NA | NA | NA | categorical | 1.57 | 1.12 | 2.73 | NA | univariable |

**^&^:**annualized percent change of weight; PFD: pirfenidone; NTD: nintedanib; %: percentage of predicted value; FVC: forced vital capacity; DLCO: diffusing lung capacity for carbon monoxide; NA: not applicable

Supplementary Table 6 Characteristics of each study about hazard ratio of weight loss predicting mortality of IPF

| **No.** | **Author** | **Time** | **Country** | **Study type** | **ntotal/ndeath** | **Age**  **(year)** | **nMale** | **nPFD/**  **NTD** | **FVC%** | **DLCO%** | **Weight loss^&^**  **type** | **HR** | **Lower limit** | **Higher limit** | **Follow-up period**  **(minimum/month)** | **Cox proportional models** |
| --- | --- | --- | --- | --- | --- | --- | --- | --- | --- | --- | --- | --- | --- | --- | --- | --- |
| 1 | Nakatsuka Y[37] | 2018 | Japan/ Britain | retrospective cohort | 210/  125 | NA | 174 | 55/NA | NA | NA | categorical(Japan)  >5% | 2.83 | 1.61 | 5.3 | 12m | univariable |
|  |  |  |  |  | 210/  125 |  |  |  |  |  | categorical(Britain)  >5% | 1.99 | 1.06 | 3.74 | 12m | univariable |
| 2 | Kim TH[18] | 2022 | South Korea | retrospective cohort | 215/  213 | 71.8±7.4 | 175 | 215/  NA | NA | NA | categorical  ≥5% | 3.27 | 2.26 | 4.74 | NA | univariable |
| 3 | Kalininskiy A[46] | 2022 | America | retrospective cohort | 71/41 | 71.3±9.5 | 50 | 17/19 | 79.2±19.4 | NA | categorical  ≥5% | 2.21 | 1.29 | 4.43 | 12m | univariable |

**^&^:**annualized percent change of weight; PFD: pirfenidone; NTD: nintedanib; %: percentage of predicted value; FVC: forced vital capacity; DLCO: diffusing lung capacity for carbon monoxide; NA: not applicable

| **Study** | **Selection** | **Comparability** | **Outcome** | **Total** |
| --- | --- | --- | --- | --- |
| Mochizuka Y 2023 | **★★★★** | **★** | **★★★** | **8★** |
| Kim TH 2022 | **★★★★** | **★** | **★★** | **7★** |
| Fujita K 2022 | **★★★★** | **★** | **★★★** | **8★** |
| Jouneau S 2022 | **★★★★** | **★** | **★★★** | **8★** |
| Zinellu A 2022 | **★★★★** | **★★** | **★★★** | **9★** |
| Yamazaki R 2022 | **★★★★** | **★** | **★★★** | **8★** |
| Gao J 2021 | **★★★★** |  | **★★****★** | **7★** |
| Kishaba T 2021 | **★★★★** |  | **★★** | **6★** |
| Suzuki Y 2021 | **★★★★** | **★** | **★★** | **7★** |
| Zinellu A 2021 | **★★★★** | **★** | **★★★** | **8★** |
| Alhamad EH 2021 | **★★★★** | **★** | **★★** | **7★** |
| Suzuki Y 2021 | **★★★★** | **★** | **★★** | **7★** |
| Alhamad EH 2020 | **★★★★** | **★** | **★★** | **7★** |
| LEE SI 2020 | **★★★★** | **★** | **★★** | **7★** |
| Aono Y 2020 | **★★★★** | **★** | **★★★** | **8★** |
| Enomoto N 2019 | **★★★★** | **★** | **★★** | **7★** |
| Jo HE 2017 | **★★★★** |  | **★★** | **6★** |
| Suzuki Y 2018 | **★★★★** | **★** | **★★** | **7★** |
| Snyder L 2019 | **★★★★** | **★** | **★★★** | **8****★** |
| Li BY 2019 | **★★★★** | **★** | **★★** | **7★** |
| Serajeddini H 2018 | **★★★★** |  | **★★★** | **7★** |
| Nishiyama O 2018 | **★★★★** | **★** | **★★★** | **8★** |
| Nakatsuka Y 2018 | **★★★★** | **★** | **★★★** | **8★** |
| Nishiyama O 2017 | **★★★★** | **★** | **★★★** | **8★** |
| Judge EP 2012 | **★★★★** | **★★** | **★★** | **8★** |
| Kondoh Y 2010 | **★★★★** | **★** | **★★★** | **8★** |
| Kondoh Y 2015 | **★★★★** | **★★** | **★★★** | **9★** |
| Bonella F 2021 | **★★★★** |  | **★★** | **6★** |
| Kim HJ 2021 | **★★★★** | **★** | **★★★** | **8★** |
| Jalaber C 2021 | **★★★★** | **★** | **★★★** | **8★** |
| Lee JS 2023 | **★★★★** | **★** | **★★★** | **8★** |
| Jouneau S 2022 | **★★★★** | **★** | **★★★** | **8★** |
| Pedraza-Serrano F 2018 | **★★★★** | **★** | **★★** | **7★** |
| Kalininskiy A 2022 | **★★★★** | **★** | **★★★** | **8★** |

Supplementary Table 7 Results of Newcastle-Ottawa quality assessment Scale for each

included studies

Supplementary Fig.1(A and B) plot for the assessment of heterogeneity among the included studies(BMI predicting mortality) through One-by-one elimination method

A: Univariate Cox hazard regression of included studies


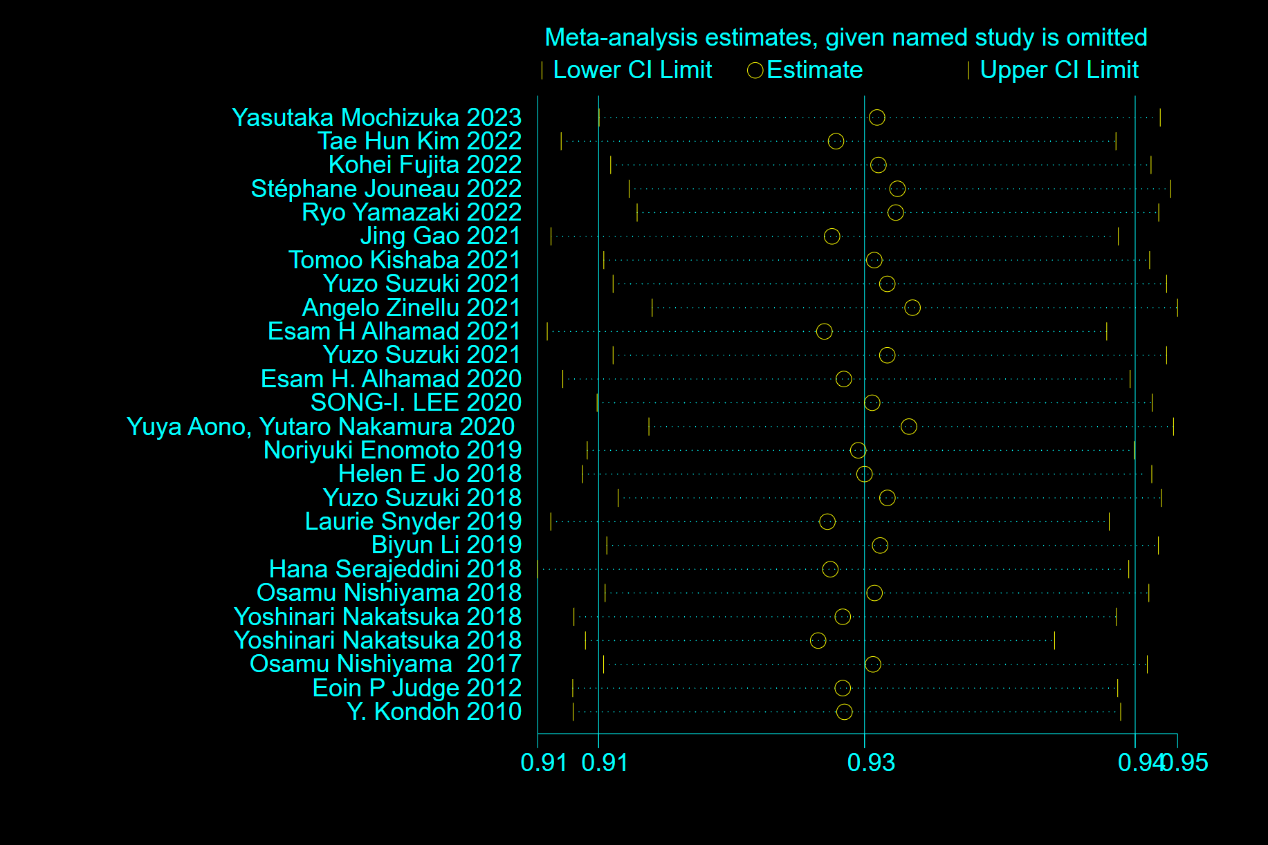


B: Multivariable Cox hazard regression of included studies


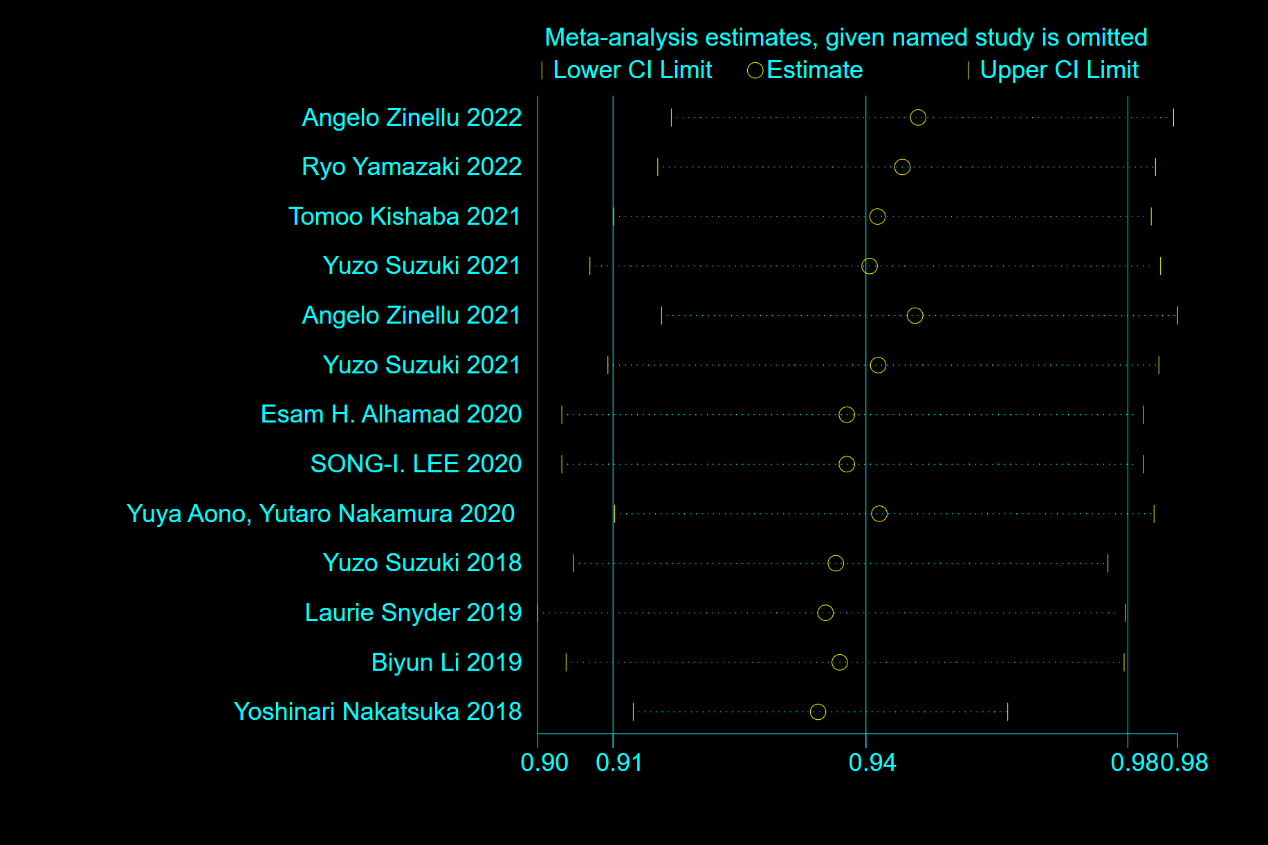


Supplementary Fig.2 plot for the assessment of heterogeneity among the included studies(BMI predicting acute exacerbation) through One-by-one elimination method


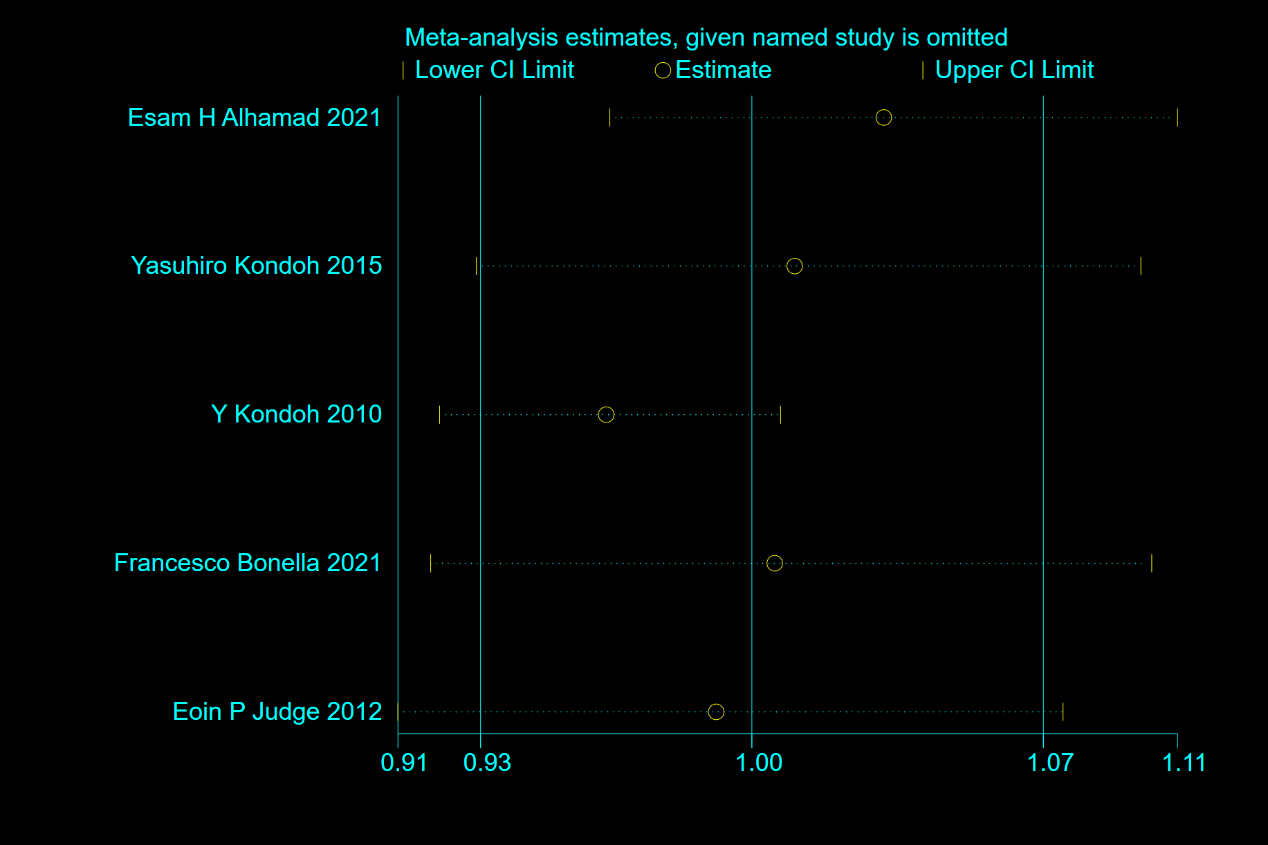


Supplementary Fig.3 plot for the assessment of heterogeneity among the included studies(BMI predicting hospitalization) through One-by-one elimination method


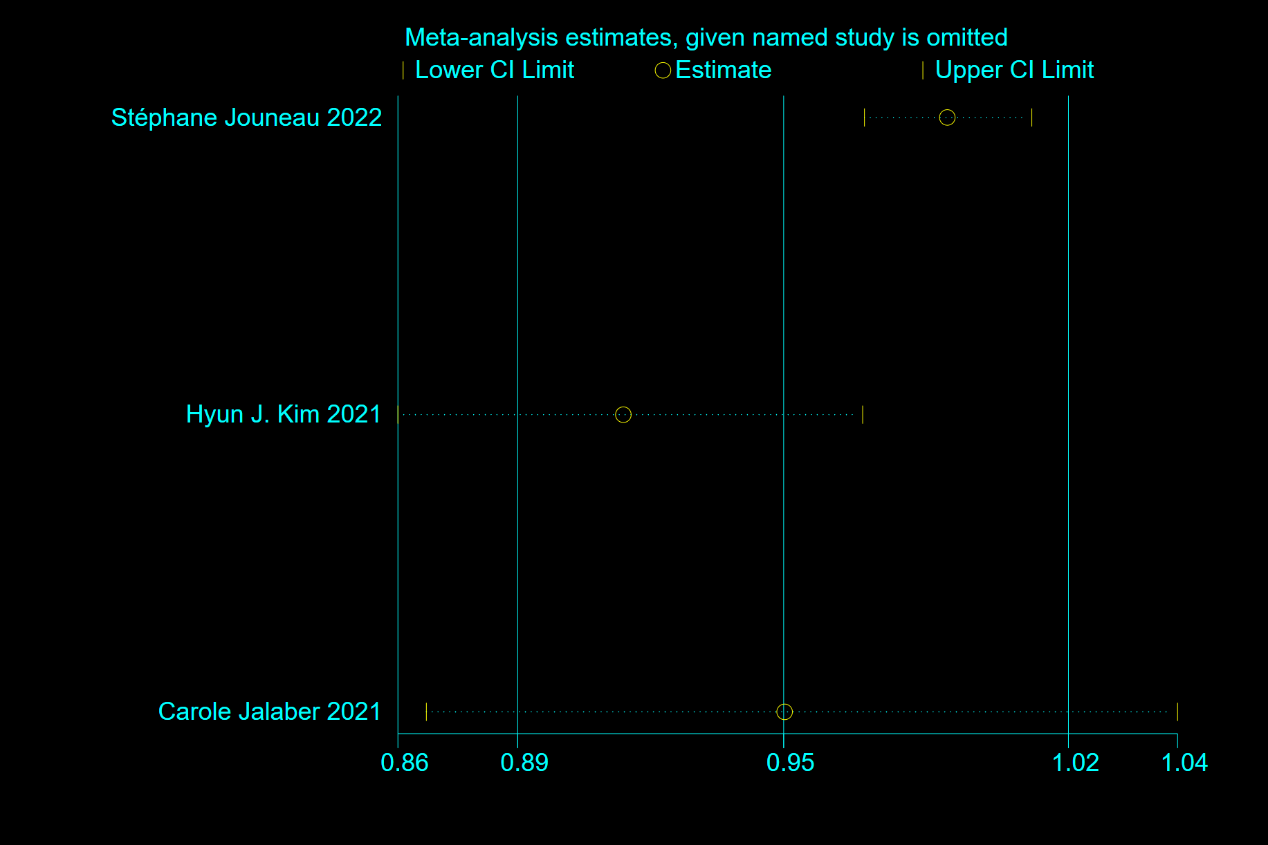


Supplementary Fig.4 plot for the assessment of heterogeneity among the included studies(HR of weight loss predicting mortality) through One-by-one elimination method


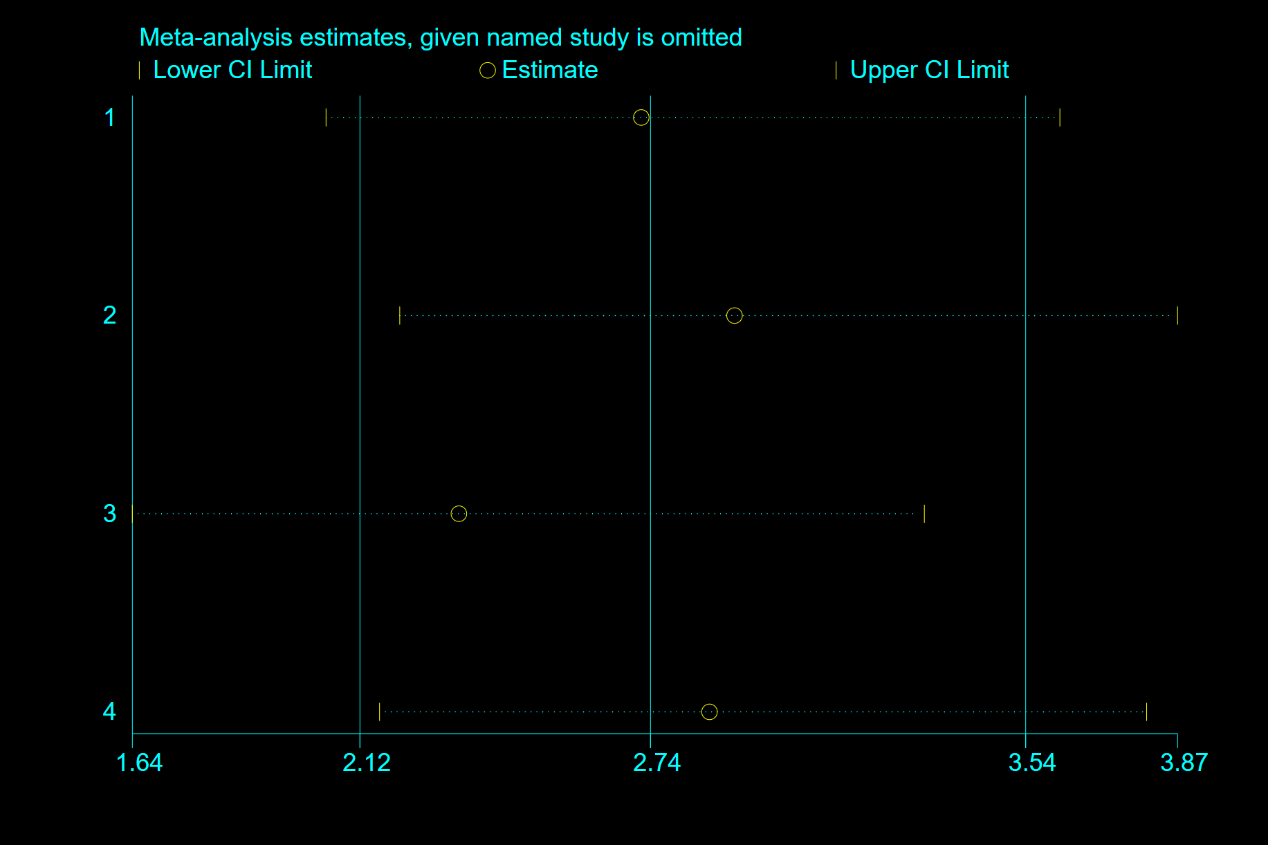


Supplementary Fig.5 plot for the assessment of heterogeneity among the included studies(OR of weight loss predicting mortality) through One-by-one elimination method


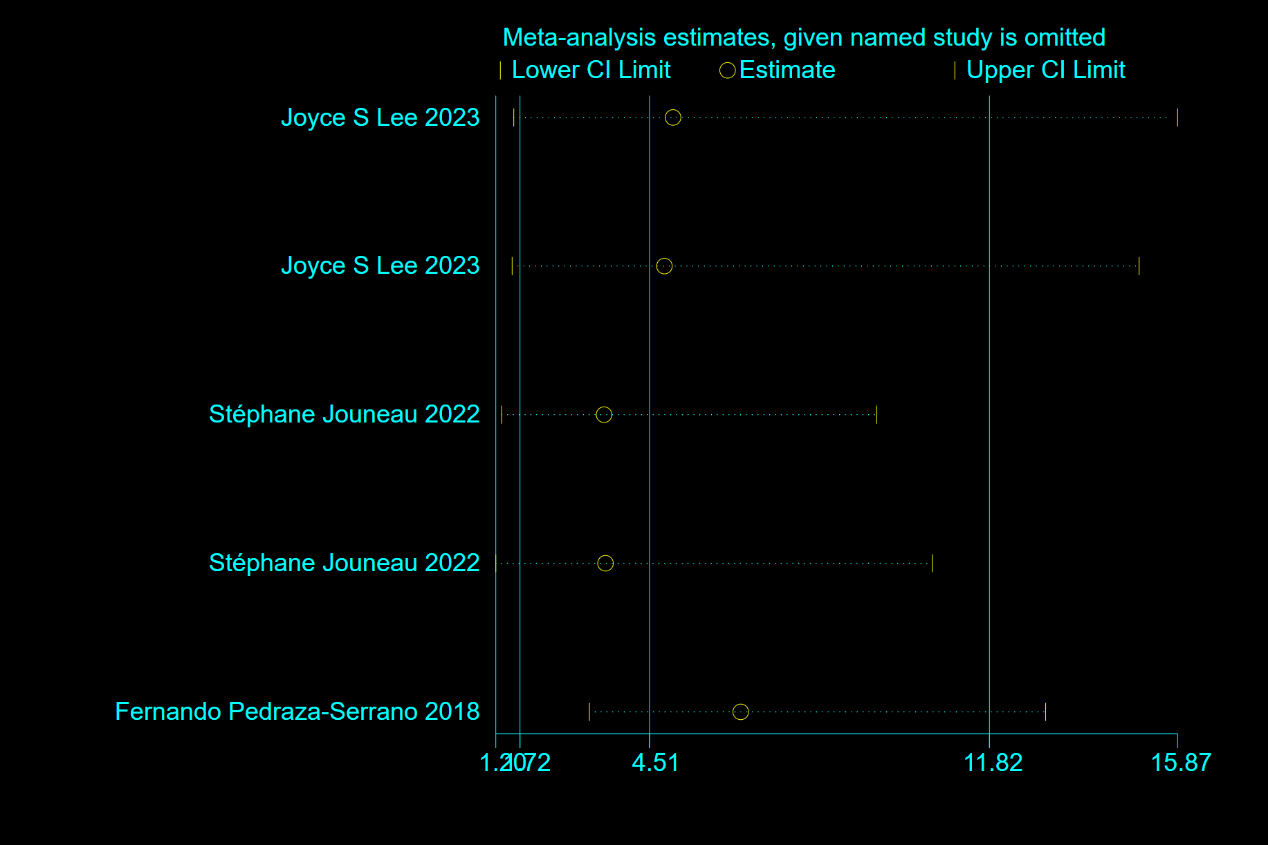

Supplement: Supplemental Material [file IANN_A_2311845_SM3802.docx]
